# Anthocyanin Characterization of Pilot Plant Water Extracts of *Delonix regia* Flowers

**DOI:** 10.3390/molecules13061238

**Published:** 2008-06-01

**Authors:** Felix Adje, Yves F. Lozano, Emmanuelle Meudec, Paul Lozano, Augustin Adima, Georges Agbo N’zi, Emile M. Gaydou

**Affiliations:** 1 CIRAD, UMR GPEB Génie des Procédés – Eau, Bioproduits, TA 40/16, 73 avenue J.F. Breton, 34398 Montpellier cedex 5, France; 2 INRA, UMR SPO Sciences pour l'Oenologie, Plateforme Polyphénols, 2 place Viala, 34060 Montpellier cedex 1, France; 3 INP-HB Institut National Polytechnique Félix Houphouët-Boigny, Laboratoire de Procédés Industriels, de Synthèse et de l’Environnement (LAPISEN), Unité Chimie de l'Eau et des Substances Naturelles, BP 1313 Yamoussoukro, Ivory Coast; 4 Université d'Abidjan-Cocody, UFR Biosciences, Laboratoire de Biochimie et Sciences des Aliments, 22 BP 582 Abidjan 22, Ivory Coast; 5 UMR CNRS 6263, Equipe AD2M (Phytochimie), Institut des Sciences Moléculaires de Marseille, Université Paul Cézanne, Faculté des Sciences et Techniques de Saint-Jérôme, Avenue Escadrille Normandie Niémen, Case 461, Marseille cedex 20, France

**Keywords:** *Delonix regia, Poinciana regia*, anthocyanin, polyphenols, extract, biodiversity

## Abstract

Following the development of new applications of pilot plant scale extraction and formulation processes for natural active bioproducts obtained from various under-utilized tropical plants and herbs, we have manufactured water-extracts from *Delonix* *regia* flowers, grown in Ivory Coast. These extracts, which contain polyphenols, are traditionally home made and used as healthy bioproducts. They are reddish-coloured due to the presence of anthocyanins. The three major anthocyanins in these extracts have been characterized. The molecular structures were confirmed by LC-SM analysis. Amongst them, two are described for the first time in *Delonix regia*.

## Introduction

Usually called flame tree, *Delonix regia*, is also known as royal *Poinciana regia* or “flamboyant”. It belongs to the Caesalpiniacea family, according to the traditional classification or to the Fabaceae family, according to the phylogenetic classification. It is considered one of the most beautiful tropical trees in the World, that produces in spring striking flame-like scarlet and yellow flowers before the leaves emerge. This tree, originally from Madagascar, but nowadays found in several countries of the intertropical zone, is often used to locally prepare extracts known to have medicinal properties [1,2]. It was reported in the literature that this plant is used in several countries to prepare extracts with antimicrobial and antifungal activities [3] and used as antibiotics [4]. In Ivory Coast, traditional medicines are prepared from several parts of the tree, including the flowers. In rural areas, water-extracts are generally home made from *Delonix* *regia* flowers. These flower extracts have never been investigated for their chemical content. The red colour of the “flamboyant” flowers is a consequence of their anthocyanin contents, that was not well investigated with modern analytical techniques to determine their molecular structures. Only few papers have reported on anthocyanin content in extracts of *Delonix* *regia* flowers [5,6] and their possible uses, such as natural pH indicators [7]. One tentative anthocyanin identification was made in 1971 on *Delonix* *regia* flower extracts, collected near Cairo [8]. That paper briefly reported that these extracts contained only two anthocyanins: cyanidin-3-glucoside and cyanidin-3-gentiobioside, which were not quantified.

## Results and Discussion

During the course of various technological developments to process natural extracts from under-used plants of the tropical biodiversity, we used pilot-plant scale membrane technology to manufacture such extracts and characterized their biomolecular compositions, their activity or their functionality [9]. 

**Figure 1:**
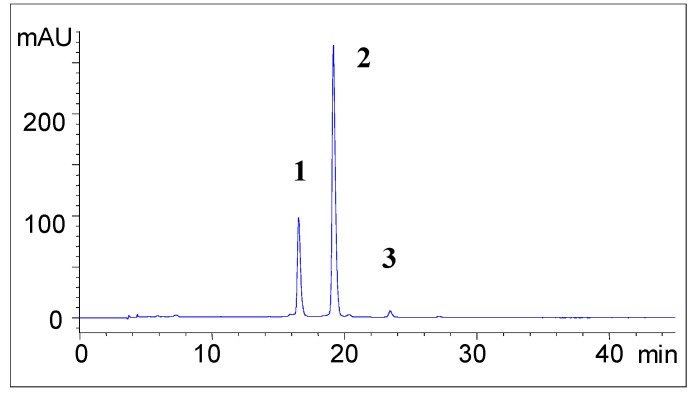
HPCL-DAD (λ 530nm) of dried *Delonix* *regia* flower extracts, macerated with acidified deionized water.

In this paper, we described the anthocyanin content of water-extract of flame tree flowers collected in Ivory Coast. Using HPLC-DAD analytical technique, we tentatively identified three major anthocyanins, as shown in Figure 1. The molecular structures of these anthocyanin compounds were confirmed on the basis of their LC-MS fragmentations (full scan MS^1^, and MS^2^) and on the shape of their UV-Vis spectra, as shown in Table 1. HPLC retention times matched with standards and confirmed the assigned molecular structures.

**Table 1:** HPLC-DAD and LC-MS data obtained from the analysis of *Delonix* *regia* flower extracts.

HPLC peak number	RT (min)	λmax (nm)	[M+H]^ +^ *m/z*	[M-X]^ +^ *m/z*	Identified anthocyanin	Ref. number
1	16.5	516	449	287 [M-162]	cyanidin 3-*O*-glucoside	[4]
2	19.1	516	595	449 [M-146] ^(a)^ 287 [M-162] ^(b)^	cyanidin 3-*O*-rutinoside	-
3	23.1	506	579	433 [M-146] ^(a)^ 271 [M-162] ^(b)^	pelargonidin 3-*O*-rutinoside	-

^(a)^: MS^1 ^fragmentation; ^(b)^: MS^2 ^fragmentation

Since HPLC-DAD analysis showed three well resolved peaks (Figure 1), it was not necessary to fractionate and to concentrate each compound before mass analysis. Direct LC-SM analysis of the extract was possible without any risk of mass fragmentation overlapping within compounds.

Investigation of the LC-MS ion fragments (Table 1) showed that the fragment at *m/z* 287 corresponds to cyanidin [10], which is a part of the molecular structures compounds **1** and **2**. UV-Vis spectra corresponding to HPLC-DAD peaks 1 and 2, showed that each compound had the same λ_max_ at 516 nm. This is also in agreement with the λ_max_ of the cyanidin aglycone [11], confirming therefore this aglycone as a part of structures of both molecules **1** and **2 **(Table 2). 

Molecular ion peaks [M+H]^+^ were *m/z* 449 for compound **1** and *m/z* 595 for compound **2**, respectively. Fragmentation (MS^1^) of compound **1** led to a loss of 162 u.m.a., and for compound **2** to a loss of 162 u.m.a. (MS^1^), then a loss of 146 u.m.a. (MS^2^). The 162 u.m.a. fragment may correspond to either a glucose or a galactose fragment. 

The *m/z* 146 fragment corresponds to a rhamnose unit. We can observe in Table 2 that fragmentation (MS^1^) never shows, on the same spectrum, both *m/z* 162 and 146 fragments. This indicates that these fragments were linked together and that they are cleaved in the sequence MS^1^, then MS^2^, from the aglycone as for a single disaccharide and not as for two monosaccharides linked to different parts of the aglycone moiety. This disaccharide should be either a rutinoside = Glc-Rha (*m/z* 308) or a Gal-Rha (*m/z* 308) linked to the cyanidin aglycone by either Glc or Rha sugar.

**Table 2:** UV-vis spectra and fragmentation analysis of *Delonix* *regia* flower extracts.

Identified Anthocyanins	HPCL-DAD UV-vis spectra	LC-MS-ESI fragmentations	
cyanidin 3-*O*-glucoside	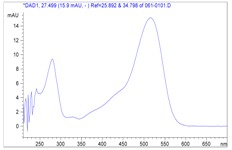	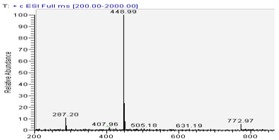	**MS^1^**
cyanidin 3-*O*-rutinoside	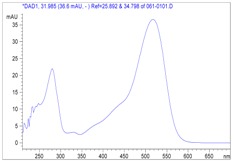	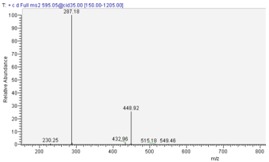	**MS^2^**
pelargonidin 3-*O*-rutinoside	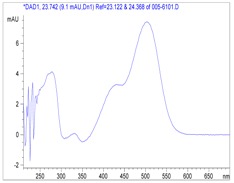	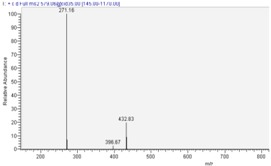	**MS^2^**

**Figure 2:**
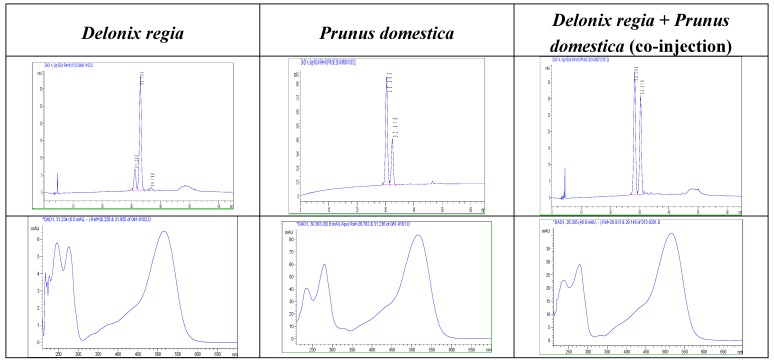
HPCL-DAD (λ 530nm) analysis of anthocyanins of acidified water-extracts of dry *Delonix* *regia* flowers and fresh *Prunus domestica* fruits and corresponding UV-vis spectra of compound **2**.

*Prunus domestica* extracts are known to contain two major anthocyanins: cyanidin 3-*O*-glucoside and cyanidin 3-*O* rutinoside [12,13]. As shown in Figure 2, the HPLC profile of the co-injection of *Delonix* *regia* and of *Prunus* *domestica* water-extracts still showed only two peaks, with increasing peak heights for both *Delonix* *regia* anthocyanins (peaks 1 and 2, Figure 1) compared to the peak heights observed for the *Delonix* *regia* extract HPLC. Thus, we may conclucde that these two extracts contained the same two anthocyanins, matched by their HPLC retention times and on chromatography confirmation using sample co-injection. 

Thus compound **1** in HPLC analysis of *Delonix* *regia* extract (Figure 1) was confirmed to be cyanidin 3-*O*-glucoside anthocyanin. Compound **2** is identified as cyanidin 3-*O*-rutinoside. 

Fragmentation spectra (MS^2^) of compound **3** (Table 2). showed the same pattern as for compound **2**. This molecule first lost (MS^1^) a mass fragment of 146 u.m.a., leading to a [M-146]^+^ mass peak (Table 1). This fragment could be attributed to a loss of a rhamnose unit. This fragment [M-146]^+^ gave, by MS^2^ fragmentation, a loss of 162 u.m.a.,, which could be a loss of glucose, leading to the [M-308]^+^ mass peak corresponding to the aglycone moiety of a *m/z* 271, identical to the pelargonidin moiety. The UV-vis spectrum of compound **3** showed a typical pattern with a λ_max_ at 506 nm and a shoulder at λ 400-450 nm, indicating the presence of a pelargonidin aglycone in the structure of this compound, in agreement with the literature [14]. Thus, compound **3** was identified as pelargonidin 3-*O*-rutinoside. Several authors have recently characterized this anthocyanin, in Moraceae fruits [15], and in *Rucus* *aculeatus* berries [16]. Their data were similar to our findings, confirming our anthocyanin identification.

Up to now, analysis of anthocyanin of acidified water-extracts of *Delonix* *regia* flowers was rarely reported in the literature. One paper reported in 1976 the presence of two anthocyanins identified as cyanidin 3-*O*-glucoside and cyanidin 3-*O*-gentiobioside, without any quantification [8]. In our study, we have only confirmed the presence of the first anthocyanin, as the second was not found (gentiobiose=Glc-Glc).

Cyanidin 3-*O*-glucoside was the second major anthocyanin (3.4 mg/L) encountered in our acidified water-extracts of dry *Delonix* *regia* flowers collected in Ivory Coast (Table 3). We identified, for the first time, two other anthocyanins, cyanidin 3-*O*-rutinoside and pelargonidin 3-*O*-rutinoside, in concentrations of 10.7 and 0.9 mg/L, respectively.

## Experimental

### Biological Material

Flowers were harvested in the centre part of Ivory Coast around the Yamoussoukro area during the blooming season. The fresh flowers were immediately dried in a ventilated oven maintained at 40°C. The dried material was packed in plastic bags, sealed under vacuum and shipped to our laboratory for pilot plant water extraction of polyphenolics.

### Extraction of anthocyanins

The extracts were obtained by soaking dried material (2.5 kg) overnight at room temperature (25°C) with deionized tap water (250 L) acidified with citric acid (0.05 N). The macerate was first filtered with a nylon cloth, then processed using a microfiltration pilot plant unit. The unit was equipped with an industrial ceramic membrane of 19 channels (60 mm diameter x 1m long), accounting for 0.304 m^2^ filtration surface. The pore size was 0.2 µm, allowing eliminating the microbial load brought by the raw material itself, without modifying the anthocyanin content of the crude extract. The red-coloured extract obtained was therefore clarified at a constant filtration flux of more than 100 L·h^-1^·m^-2^·b^-1^ for several hours. This microfiltrated extract was subject to anthocyanin analysis and underwent to further process steps to prepare polyphenol concentrated extracts of *Delonix* *regia* flowers in liquid or powder forms.

**Table 3:** Anthocyanin content in the microfiltrated water-extract of *Delonix* *regia* flowers.

Anthocyanins	Molecular structures	mg/L ^a^
cyanidin 3-*O*-glucoside	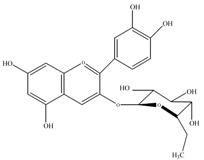	3.4±0.2
cyanidin 3-*O*-rutinoside	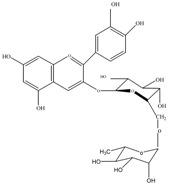	10.7±0.6
pelargonidin 3-*O*-rutinoside	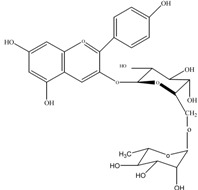	0.9±0.1

a: as cyanidin equivalents (MW=287), means of triplicate analysis

### HPLC-DAD analyses.

Anthocyanins were analyzed by HPLC using a diode array detector (Agilent Technologies, 1100 series, France). The detection was set at λ 530 nm for anthocyanins. The separation column was a RP 18 column (Satisfaction column, 250 mm x 4.6 mm, 0.45µm, Cil Cluzeau, France). The column temperature was maintained at 30°C using a controlled-temperature oven. The binary solvent system was composed of 10:90 formic acid/water (solvent A) and 10:90 formic acid/acetonitrile (solvent B). The linear solvent gradient started with an initial mobile phase of 95% A and 5% B, to a mobile phase of 62% A and 38% B during 55 min. The washing cycle of the column used a mixture of acetonitrile/water (50/50) for 10 min. The flow rate for both analysis and washing cycles was set at 0.8 mL·min^-1^. For specific identification of cyanidin 3-*O* glucoside and cyanidin 3-*O* rutinoside anthocyanins with HPLC-DAD co-elution of *Delonix* *regia* and *Prunus* *domestica* extracts, the elution was made with a linear gradient of A (formic acid/water, 0.5/99.5) and B (formic acid/acetonitrile 0.5/99.5): from 95% A - 5% B, to 60% A - 40% B within 60 min, at 0.8 mL·min^-1^.

### HPLC-MS analyses.

The HPLC-MS-ESI analytical technique was used to confirm the chemical structures and the identities of anthocyanin molecules considered in this study. The molecules were analyzed by HPLC equipped with a DAD detector (Waters-Alliance 2690) on a Merck LiChrospher 100-RP 18 column (250 x 2 mm, 5µm pore size), coupled with an ion trap mass spectrometer (LCQ-Advantage, Thermo Electron S.A., Courtaboeuf, France). The mobile phase consisted of (A) water and formic acid (98:2, v/v) and (B) water, acetonitrile and formic acid (18:80:2, v/v). The gradient method started at 0.25 mL·min^-1^ from 94% to 50% (A) over 55 min. The heated capillary and voltage was maintained at 175°C and 2 kV, respectively. The full-mass scan spectra from *m/z* 100 to 2000 were collected. All mass spectrometry data were acquired with a positive ionization mode.
